# The application of human periodontal ligament stem cells and biomimetic silk scaffold for in situ tendon regeneration

**DOI:** 10.1186/s13287-021-02661-7

**Published:** 2021-12-04

**Authors:** Jialin Chen, Qingyun Mo, Renwang Sheng, Aijing Zhu, Chen Ling, Yifan Luo, Aini Zhang, Zhixuan Chen, Qingqiang Yao, Zhuoying Cai, Wei Zhang

**Affiliations:** 1 School of Medicine, Southeast University, Nanjing, 210009 China; 2 Jiangsu Key Laboratory for Biomaterials and Devices, Southeast University, Nanjing, 210096 China; 3 China Orthopedic Regenerative Medicine Group (CORMed), Hangzhou, China; 4 Department of Orthopaedic Surgery, Institute of Digital Medicine, Nanjing First Hospital, Nanjing Medical University, Nanjing, 210006 China; 5 Oral and Maxillofacial Surgery, The Second Affiliated Hospital of Zhejiang University, Hangzhou, 310009 China

**Keywords:** PDLSCs, Silk, Biomimetic scaffold, Tendon repair, Tissue regeneration

## Abstract

**Background:**

With the development of tissue engineering, enhanced tendon regeneration could be achieved by exploiting suitable cell types and biomaterials. The accessibility, robust cell amplification ability, superior tendon differentiation potential, and immunomodulatory effects of human periodontal ligament stem cells (hPDLSCs) indicate their potential as ideal seed cells for tendon tissue engineering. Nevertheless, there are currently no reports of using PDLSCs as seed cells. Previous studies have confirmed the potential of silk scaffold for tendon tissue engineering. However, the biomimetic silk scaffold with tendon extracellular matrix (ECM)-like structure has not been systematically studied for in situ tendon regeneration. Therefore, this study aims to evaluate the effects of hPDLSCs and biomimetic silk scaffold on in situ tendon regeneration.

**Methods:**

Human PDLSCs were isolated from extracted wisdom teeth. The differentiation potential of hPDLSCs towards osteo-, chondro-, and adipo-lineage was examined by cultured in different inducing media. Aligned and random silk scaffolds were fabricated by the controlled directional freezing technique. Scaffolds were characterized including surface structure, water contact angle, swelling ratio, degradation speed and mechanical properties. The biocompatibility of silk scaffolds was evaluated by live/dead staining, SEM observation, cell proliferation determination and immunofluorescent staining of deposited collagen type I. Subsequently, hPDLSCs were seeded on the aligned silk scaffold and transplanted into the ruptured rat Achilles tendon. Scaffolds without cells served as control groups. After 4 weeks, histology evaluation was carried out and macrophage polarization was examined to check the repair effects and immunomodulatory effects.

**Results:**

Human PDLSCs were successfully isolated, and their multi-differentiation potential was confirmed. Compared with random scaffold, aligned silk scaffold had more elongated and aligned pores and promoted the proliferation and ordered arrangement of hPDLSCs. After implantation into rat Achilles tendon defect, hPDLSCs seeded aligned silk scaffold enhanced tendon repair with more tendon-like tissue formation after 4 weeks, as compared to the scaffold-only groups. Higher expression of CD206 and lower expression of iNOS, IL-1β and TNF-α were found in the hPDLSCs seeded aligned silk scaffold group, which revealed its modulation effect of macrophage polarization from M1 to M2 phenotype.

**Conclusions:**

In summary, this study demonstrates the efficacy of hPDLSCs as seed cells and aligned silk scaffold as a tendon-mimetic scaffold for enhanced tendon tissue engineering, which may have broad implications for future tendon tissue engineering and regenerative medicine researches.

**Supplementary Information:**

The online version contains supplementary material available at 10.1186/s13287-021-02661-7.

## Background

Tendon is a dense connective tissue, which connects muscle to bone and therefore plays important roles in the force transmission of the human musculoskeletal system. During the past decades, the occurrence of tendon injury is keeping increasing and brings a huge burden on the global economy. There are at least 30 million tendon injuries annually, which costs more than $30 billion per year [1, 2]. Tendon healing in adults is unsatisfactory, usually with scar tissue formation and re-rupture occurrence. The development of tissue engineering makes it possible for tendon regeneration after injury. Various cell types have been applied as seed cells for tendon tissue engineering, and different natural or synthetic biomaterials have been used as scaffolds to construct tissue-engineered tendon [3–5]. Although promising results have been shown in tendon healing models of animals, the search for the combination of ideal seed cells and suitable scaffolds is still ongoing.

Human periodontal ligament stem cells (hPDLSCs), first identified in 2004, were isolated from the periodontal ligament of surgically extracted human third molars (i.e., wisdom tooth) [6]. Similar to bone marrow MSCs (BMSCs), PDLSCs express MSC surface markers and have the capacity of clone formation and differentiation potential to osteoblasts, chondrocytes and adipocytes [6, 7]. The accessibility and immunomodulatory effects of PDLSCs make them being one of the ideal seed cells for tissue engineering [7, 8], and PDLSCs have been used in the construction/repair of some tissues including periodontal tissue [9], bone [10], cornea [11] and nerve [12]. Since ligament and tendon share high similarities in tissue structure and function, it is reasonable to speculate that PDLSCs could possess unique advantages in tendon tissue engineering, as compared to other adult tissue stem cells. Moshaverinia et al. compared the tendon regenerative capacity among PDLSCs, gingival mesenchymal stem cells (GMSCs) and BMSCs. Results showed that PDLSCs had significantly greater capacity than either GMSCs or BMSCs [13]. Nevertheless, to our knowledge, there are no reports of using PDLSCs as seed cells in current tendon tissue engineering. Therefore, it is necessary to further evaluate the efficacy of PDLSCs for tendon repair and regeneration in more circumstances.

The extracellular matrix (ECM) in the native tendon is composed of aligned collagen fibres. To mimic the physiological structure of the native tendon, aligned scaffolds made from different materials have been developed, including polylactic acid (PLA) [14], poly(ε-caprolactone) (PCL) [15], chitosan [16], collagen [17], and so on. Different materials have their advantages and disadvantages when used as scaffolds for tendon tissue engineering. Generally, the application of synthetic polymers is limited by their relatively poor integration ability with host tissues, as well as the side effects (e.g., inflammatory response) of degradation products in vivo [18, 19]. Collagen is the main ECM component of the native tendon and therefore has excellent biocompatibility. However, the cost of collagen extraction is high and the degradation is relatively rapid (could be improved by cross-linking to some extent), which is not ideal for tendon tissue engineering as tendon has poor self-repair ability and requires a long healing duration [3]. In contrast, silk could be easily obtained at a low cost, possessing excellent mechanical properties and a relatively slow rate of controllable degradation. Lots of previous studies have confirmed the potential of silk as an ideal scaffold material for tendon tissue engineering, with prospects for clinical application [20–22]. However, the biomimetic silk scaffold with tendon ECM-like structure has not been systematically studied for in situ tendon regeneration.

To address these concerns, this study aims to evaluate the effects of hPDLSCs and biomimetic silk scaffold on tendon tissue engineering and regeneration. Human PDLSCs were isolated from extracted wisdom teeth, and their multi-differentiation potential was confirmed. Silk scaffolds with aligned and random silk scaffold structures were fabricated by the controlled directional freezing technique, and their physicochemical and cytocompatibility properties were evaluated. Finally, a tissue-engineered tendon was constructed with characterized hPDLSCs and biomimetic silk scaffold and transplanted into rat Achilles tendon defect model. After 4 weeks of implantation, histology evaluation was carried out and macrophage polarization was examined to evaluate the repair effects and immunomodulatory effects.

## Methods

### Isolation and culture of hPDLSCs

Human PDLSCs were isolated from periodontal ligaments of surgically extracted human third molars. The study protocol was following the principles of the Declaration of Helsinki and approved by the Ethics Committee of the Second Affiliated Hospital of Zhejiang University (I20200011193), with written informed consent obtained from all three patients 20–40 years of age. All patients had no other pathological conditions. To isolate hPDLSCs, the periodontal ligaments were collected and cut into 1–2 mm^3^ pieces and digested overnight in 4 ml collagenase type I (Biofroxx, 1904MG100) at a concentration of 2.5 mg/ml. After centrifugation and resuspension, cells and undigested tissue fractions were seeded in a 10 cm dish (NEST Biotechnology, 704001) at a low density (at most 1000 cells) in low glucose DMEM (Gibco, C11885500) supplemented with 10% fetal bovine serum (Wisent, 086-550) and 1% penicillin–streptomycin (Gibco, 15140122). The colonies that formed after around 10 days of culture were digested with trypsin and passaged. Cells between passages 3 and 6 were used in the following experiments. Cell experiments were repeated in PDLSCs derived from different patients.

### Multi-lineage differentiation

The differentiation potential of hPDLSCs towards osteo-, chondro-, and adipo-lineage was examined using previously established protocols [23, 24]. Briefly, for osteogenic differentiation, cells were cultured in the inducing medium for one week, and alkaline phosphatase (ALP) was stained (Beyotime, C3206). Human PDLSCs were also cultured in the osteogenic differentiation medium for two weeks and then stained with 1% alizarin red S (ARS, pH 4.2; Solarbio, G1452). For chondrogenic differentiation, cells were induced in a micromass culture system for two weeks and Alcian Blue staining (Macklin, A801642) was performed. For adipogenic differentiation, cells were cultured in the inducing medium for three weeks and stained with Oil Red O (Solarbio, G1260).

### Fabrication of aligned and random silk scaffold

The scaffolds were prepared as previously reported [25]. Briefly, 6% (w/v) silk fibroin solution was obtained by extraction, dissolved, and dialyzed and then put into a polystyrene cylinder tube (12 mm diameter × 52 mm height). To obtain aligned scaffolds, the bottom of the tube was attached to a precooled aluminium plate (− 80 °C). And the lateral of the tube was insulated by sponges. To obtain random scaffolds, the whole tube was insulated by sponges. These sets were then placed at − 80 °C overnight to control the growth direction of ice crystals in silk solution to form the aligned and random scaffolds. Subsequently, the scaffolds were lyophilized (Boyikang, FD-1A-50) and treated with 90% methanol (v/v, Sinopharm). The scaffolds were re-lyophilized and cut into the required size for the following experiments.

### Scanning electron microscopy (SEM) observation

The samples were mounted on stubs and coated with gold. A Zeiss EVO 18 SEM (Carl-Zeiss) was then used to observe the surface structure of the samples. For the quantification of the pore architectures of scaffolds, in total 50 pores of each group were randomly selected, and the area and the aspect ratio of these pores were determined by using ImageJ analysis software (NIH).

### Water contact angle determination

A contact angle system (JC2000D, Shanghai Zhongchen) was used to determine the water contact angles of the scaffolds under 50% relative humidity at 37 °C, which recorded the gas-liquid-solid interface after dropping one droplet (5 μl) of deionized water onto the scaffolds. The contact angles were then measured based on the videos.

### Swelling ratio determination

The swelling ratio of the scaffolds (*n* = 4 per group) was determined as previously described [26]. Briefly, the weight of the scaffolds was determined before (dry weight, W0) and after immersed in PBS for a designated time (W1). The swelling ratio was calculated using the formula: Swelling ratio = (W1 − W0)/W0.

### Degradation of scaffolds in vitro

The weight of the scaffolds was determined (dry weight, W0; *n* = 5 per group) at day 0 and then immersed in PBS with shaking at 37 °C. At each designated time point, the scaffolds were washed, dried, and weighed (W1). The weight remaining was calculated using the formula: Weight remaining (%) = W1/W0*100.

### Mechanical testing

Mechanical testing was performed using an electric universal testing machine (UTM2502; Sunstest, Shenzhen, China) at an elongation rate of 1 mm/min. The structural properties of the random and aligned scaffolds (*n* = 5 per group) in wet condition were represented by ultimate load (N) and Young’s modulus (kPa).

### Live/dead staining

Human PDLSCs (8 × 10^4^ cells/scaffold) were seeded on aligned and random scaffolds (15 mm × 7.5 mm × 0.5 mm, *L* × *W* × *H*) in a nonadherent 24-well plate and cultured in the growth medium. On day 7, samples were incubated with a working solution of calcein-AM/PI Double Staining Kit (Dojindo, C542) for 20 min in a 37 °C incubator. The cells were then observed under a fluorescence microscope.

### Cell proliferation on scaffolds

Human PDLSCs (2.2 × 10^4^ cells/scaffold) were seeded on aligned and random scaffolds (10 mm long × 6 mm wide) in a nonadherent 48-well plate and cultured in the growth medium for 3 and 7 days. At the designated time points, cells were incubated with Cell Counting KIT-8 (CCK-8, ApexBio, k1018) solution for one hour in a 5% CO_2_ incubator. The produced formazan was measured using an 800 TS microplate reader (BioTek) at 450 nm.

### Immunofluorescence

Human PDLSCs (8 × 10^4^ cells/scaffold) were seeded on aligned and random scaffolds (15 mm × 7.5 mm × 0.5 mm, *L* × *W* × *H*) in a nonadherent 24-well plate. The constructs were cultured in high glucose DMEM (Gibco, C11995500) supplemented with 10% fetal bovine serum, 1% penicillin–streptomycin and 50 μg/ml L-ascorbic acid 2-phosphate (A2-P; Sigma-Aldrich, A8960). After 7 days of culture, samples were collected, fixed, permeabilized and blocked. Subsequently, samples were incubated with rabbit anti-collagen type I (COL I) primary antibody (Proteintech, 14695-1-AP) at 4 °C overnight. After washing, samples were incubated with 488-conjugated goat anti-rabbit IgG (Proteintech, SA00013-2) for 1 h at room temperature. Finally, 4′,6-diamidino-2-phenylindole (DAPI; Beyotime, C1002) was used to reveal the nuclei of the cells.

### Animal experiment

Five adult female Sprague–Dawley rats weighing 200–220 g were used. The study protocol was approved by the animal experimental ethics committee of Southeast University (20191015016). Before the operation, all the rats were treated with 150 mg/kg cyclophosphamide for 24 h. Under general anaesthesia, a gap of Achilles tendon defect with 6 mm in length was created. Scaffolds (10 mm × 6 mm × 1 mm, *L* × *W* × *H*) with or without 5 × 10^5^ hPDLSCs seeded were sutured to the remaining tendon tissues (Fig. 4A, *n* = 3 for each group). The skin was subsequently closed, and the rats were allowed free cage activity after surgery. After 4 weeks, specimens were harvested for the following evaluations.

### Histological examination

Histological examination was performed as previously described [20]. Briefly, the collected specimens were fixed, dehydrated, and embedded in paraffin. The paraffin blocks were then sectioned by a microtome. Subsequently, haematoxylin and eosin (H&E) staining and Masson trichrome staining were carried out by following standard procedures. To quantify the repair effect of injured tendons, histological scoring was done based on six parameters of H&E staining pictures, which includes fibre structure, fibre arrangement, nuclear roundness, vascularity, inflammation, and cell quantity. Picrosirius red staining was also performed and observed under polarized light microscopy to evaluate the distribution of collagens.

### Immunohistochemistry

Immunohistochemical staining was performed as previously described [25]. The primary antibodies used in this study were mouse anti-human nuclei monoclonal antibody (Sigma-Aldrich, MAB1281), anti-COL I polyclonal antibody (Servicebio, GB11022-3), anti-CD206 polyclonal antibody (Servicebio, GB13438), anti-inducible nitric oxide synthase (iNOS) polyclonal antibody (Servicebio, GB11119), anti-IL-1β polyclonal antibody (Servicebio, GB11113), and anti-TNF-α polyclonal antibody (Servicebio, GB13452). To quantify the results of immunohistochemistry, representative images were analysed by ImageJ. For COL I, IL-1β and TNF-α, the positive area and total area of images were measured and the ratio was calculated by positive area/total area. For COL I, integrated optical density (IOD) values of the positive area were determined. For CD206 and iNOS, the positive cells and total cells of images were counted and the ratio was calculated by positive cells/total cells.

### Statistical analysis

All data are presented as mean ± SD. Student’s t test was used for the comparison between two groups. One-way ANOVA with Bonferroni post hoc correction was used in experiments with more than two groups. Experiments were repeated, and representative results are shown. For all comparisons, p value below 0.05 was considered statistically significant.

## Results

### Human PDLSCs possess self-renew and multi-lineage differentiation potential

**Fig. 1:**
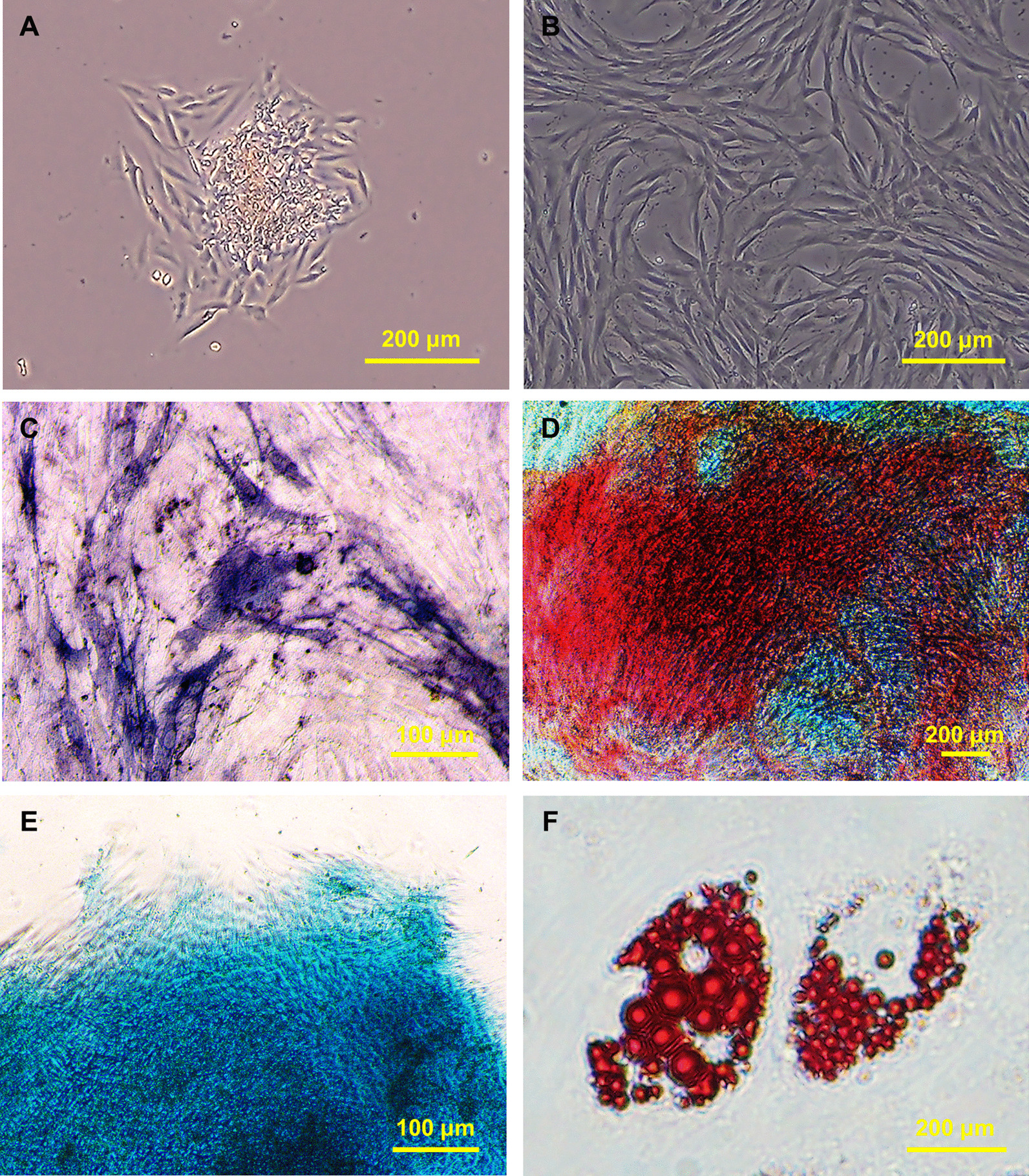
Isolation and characterization of hPDLSCs. **A** Clone formation at around 10 days of culture. **B** Spindle cell morphology of hPDLSCs. **C** Alkaline phosphatase (ALP) staining after osteogenic differentiation for one week. **D** Alizarin red S (ARS) staining after osteogenic differentiation for two weeks. **E** Alcian Blue staining after chondrogenic differentiation for two weeks. **F** Oil Red O staining after adipogenic differentiation for three weeks

Human PDLSCs were isolated as the protocols described above. Clone formation was observed at around 10 days of culture after seeding (Fig. 1A). After passage, a spindle cell morphology was shown, which is similar to fibroblast (Fig. 1B). To determine the multi-lineage differentiation potential of hPDLSCs, cells were induced towards osteo-, chondro-, and adipo-lineage with the specific inducing media. Osteogenic differentiation assays showed the existence of alkaline phosphatase (Fig. 1C) and calcium deposits (Fig. 1D). Alcian Blue staining confirmed the chondrogenic differentiation potential of hPDLSCs (Fig. 1E). In addition, oil red staining showed the accumulation of lipid droplets after induction, which confirmed the adipogenic differentiation potential of hPDLSCs (Fig. 1F).

### Scaffold fabrication and characterization

**Fig. 2:**
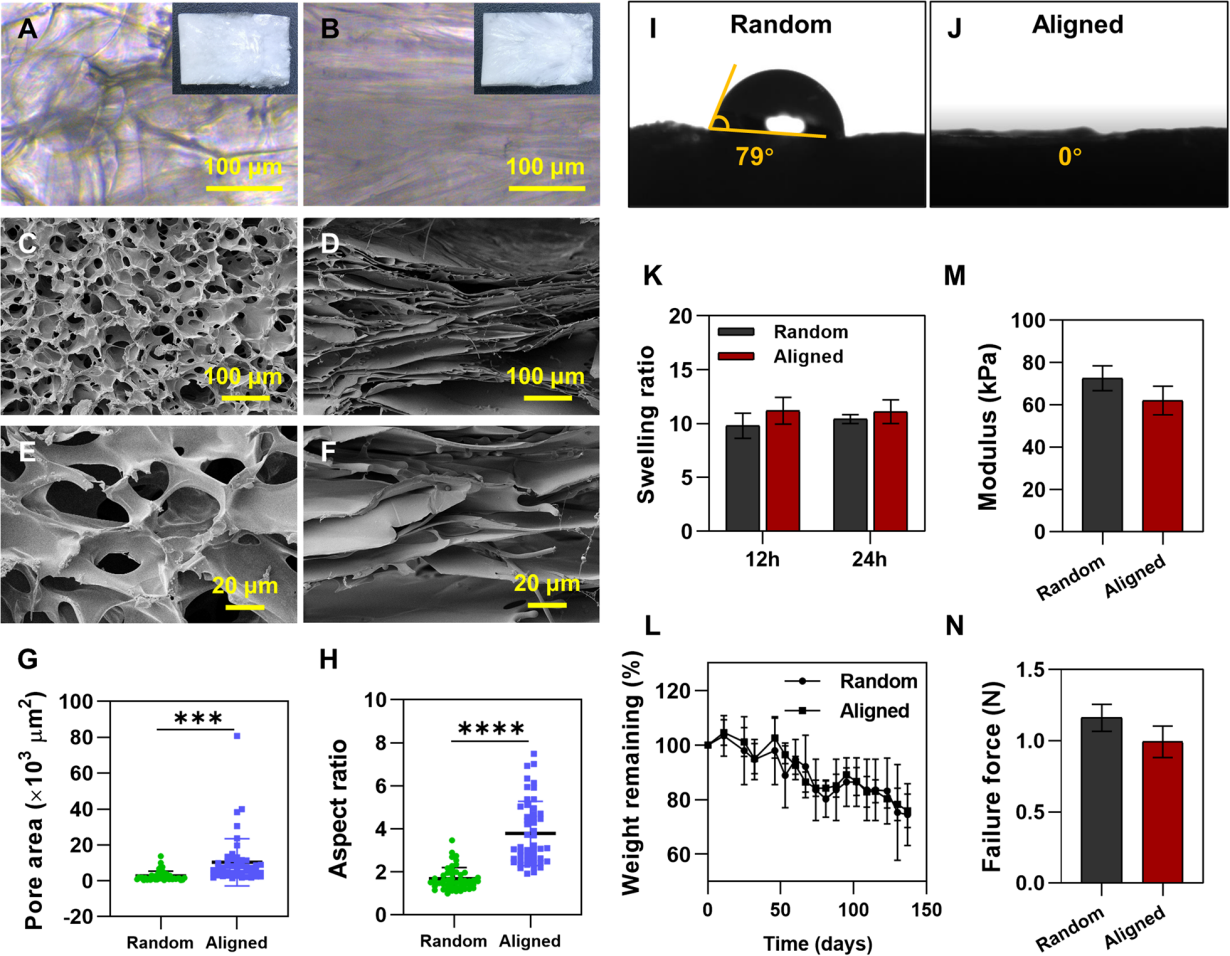
Fabrication and characterization of silk scaffolds. Fabricated silk scaffolds were visualized under light microscopy. **A** Random scaffold. **B** Aligned scaffold. The upper right panels of **A** and **B** indicate the gross morphology of each scaffold. Scaffolds were also observed under SEM with different magnifications. **C**, **E** Random scaffold. **D**, **F** Aligned scaffold. Pore area (**G**) and aspect ratio (**H**) were quantified by ImageJ. **I**, **J** Water contact angles of silk scaffolds at 1 s after dropping the water. **K** Swelling ratios of silk scaffolds. **L** In vitro degradation curves of the scaffolds in PBS at 37 °C. **M**, **N** Mechanical properties of random and aligned scaffolds. ***p < 0.001; ****p < 0.0001

Random and aligned silk scaffolds were fabricated (upper right panels of Fig. 2A, B). The pore architectures were visualized under light microscopy (Fig. 2A, B) and SEM (Fig. 2C–F). In the random group, pores were disorganized. While in the aligned group, long and thin pores were aligned along the direction of temperature gradient during directional freezing. The area of pores in the aligned silk scaffold (10.39 ± 13.18 × 10^3^ μm^2^) was higher than that in the random silk scaffold (2.69 ± 2.65 × 10^3^ μm^2^) (Fig. 2G). A significantly increased aspect ratio was also found in the aligned group (3.79 ± 1.50), as compared to the random group (1.68 ± 0.52) (Fig. 2H). From the morphology evaluation of prepared scaffolds, it was found that random silk scaffolds possessed more homogeneous but randomly directed pores, while aligned silk scaffolds possessed more elongated and aligned pores.

The prepared scaffolds were further characterized. Water contact angle determination of each type of scaffold showed that the aligned silk scaffold was more hydrophilic than the random scaffold. The contact angle of the random scaffold was 79° at 1 s after dropping the water (Fig. 2I). In contrast, it took less than 1 s for the water penetration in the aligned silk scaffold (Fig. 2J). Nevertheless, no significant difference was found between the two groups on the swelling ratio after scaffolds being immersed in PBS for 12 and 24 h (Fig. 2K). The scaffolds were immersed in PBS with shaking at 37 °C for an even longer time (up to 137 days) to detect their degradation in vitro. Comparable degradation speed was found between two kinds of scaffolds (Fig. 2L). On day 137, the weight remaining of random scaffolds was 74.52 ± 11.34%, as compared to 75.89 ± 6.13% in aligned scaffolds. The mechanical properties of scaffolds were also tested and compared. Similar values of modulus and failure force were found (Fig. 2M, N).

### Silk scaffolds regulate cell arrangement and behaviours in vitro

**Fig. 3:**
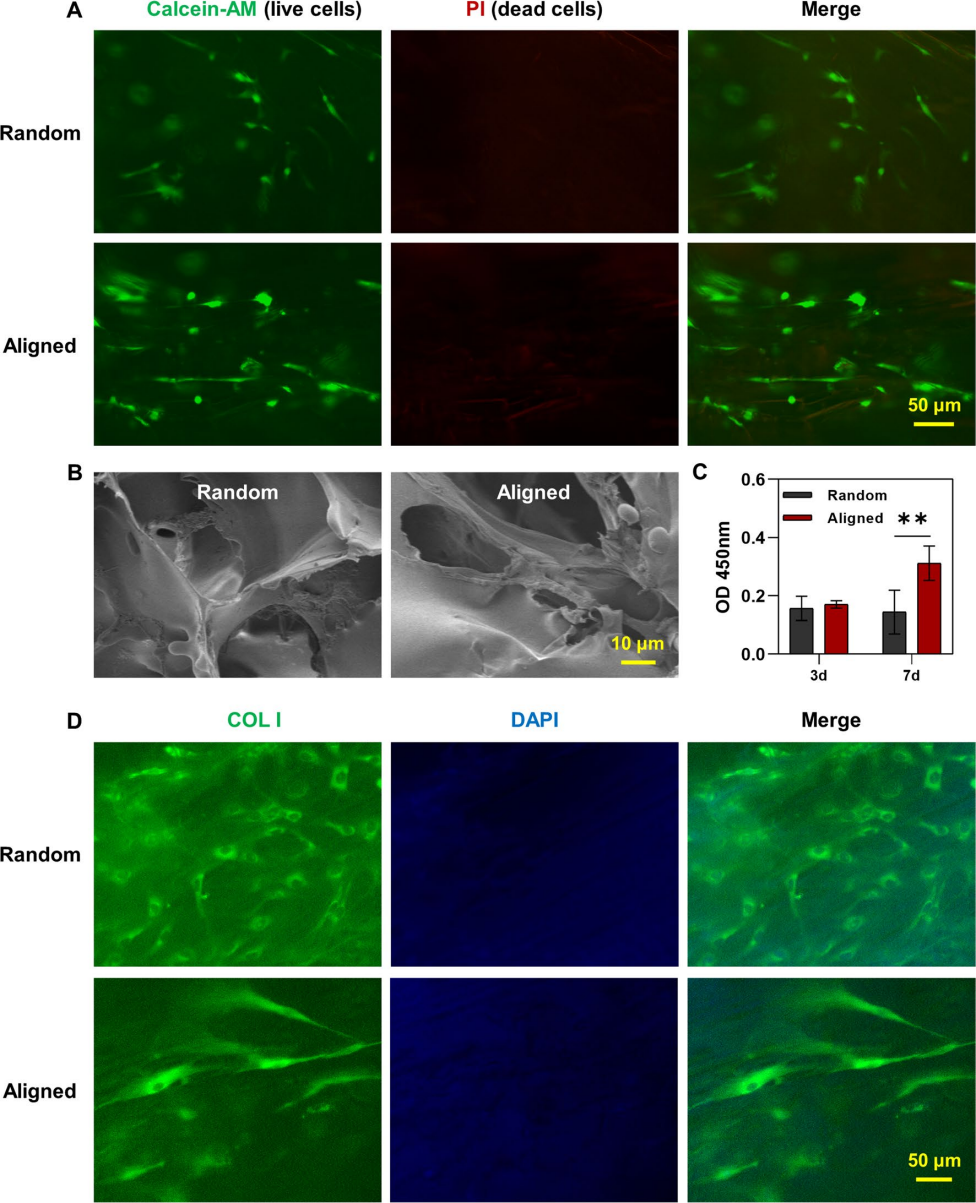
Cell-scaffold interaction in vitro. **A** Live/dead staining of hPDLSCs after 7 days of culture on random and aligned silk scaffolds. Viable cells were stained by calcein-AM (green) and dead cells were stained by PI (red). **B** Cell seeded scaffolds were observed under SEM. **C** Proliferation of hPDLSCs cultured on silk scaffolds for 3 and 7 days, which was measured by CCK-8. **D** Human PDLSCs were seeded on scaffolds and cultured in the tenogenic differentiation medium for one week. The expression of COL I was evaluated by immunofluorescence staining. **p < 0.01

To evaluate the cell-scaffold interaction, hPDLSCs were seeded on silk scaffolds and their cell behaviours were examined (Fig. 3). Although scaffolds showed strong autofluorescence, it was noticed that cells distributed through the whole silk scaffolds due to their three-dimensional growth microenvironment. Live/dead staining revealed that the seeded hPDLSCs were remained viable after 7 days of culture and no dead cells could be found (Fig. 3A). More cell alignment was shown in the aligned silk scaffold as compared to that in the random scaffold. Besides, pronounced elongated cell morphology was found in the aligned group with SEM imaging (Fig. 3B). To evaluate the proliferation of cells cultured on scaffolds, CCK-8 was used and the results showed that cells grew faster on the aligned scaffold at day 7, as compared to the random group (Fig. 3C). Human PDLSCs were seeded on scaffolds and cultured in the tenogenic differentiation medium for one week. The deposition of COL I was found in both groups, with a more aligned arrangement on the aligned silk scaffold (Fig. 3D). Gene expression of tendon-related genes including Scleraxis (*SCX*), Mohawk (*MKX*), *COL I*, EPH receptor A4 (*EPHA4*), Nuclear factor of activated T-cells 4 (*NFATC4*) and Biglycan (*BGN*) was evaluated by qPCR. However, no significant difference was found between groups (Additional file 1: Figure S1).

### The combination of hPDLSCs and aligned silk scaffold promotes tendon repair in vivo

**Fig. 4:**
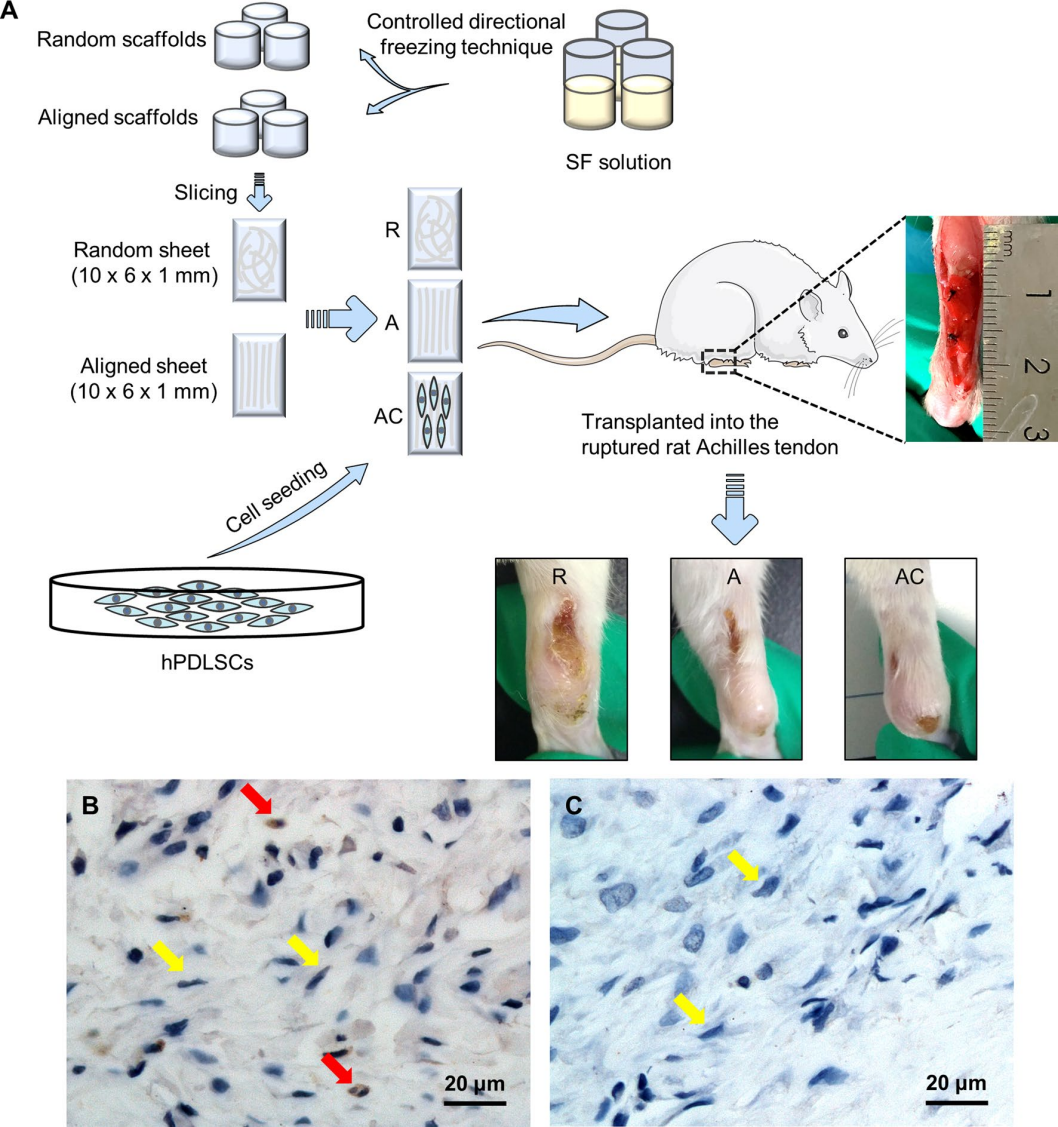
Animal experiment and tracing of implanted cells. **A** Schematic illustration of animal model and grouping, as well as the gross morphology of repaired rat Achilles tendon. R group, random silk scaffold alone. A group, aligned silk scaffold alone. AC group, hPDLSCs seeded aligned silk scaffold. **B** Immunohistochemistry with anti-human nuclei monoclonal antibody. Red arrows indicate the transplanted human cells, nuclei of which were dyed by both haematoxylin and DAB. Yellow arrows indicate the nuclei of rat cells dyed by haematoxylin only. **C** Negative control of immunohistochemistry

**Fig. 5:**
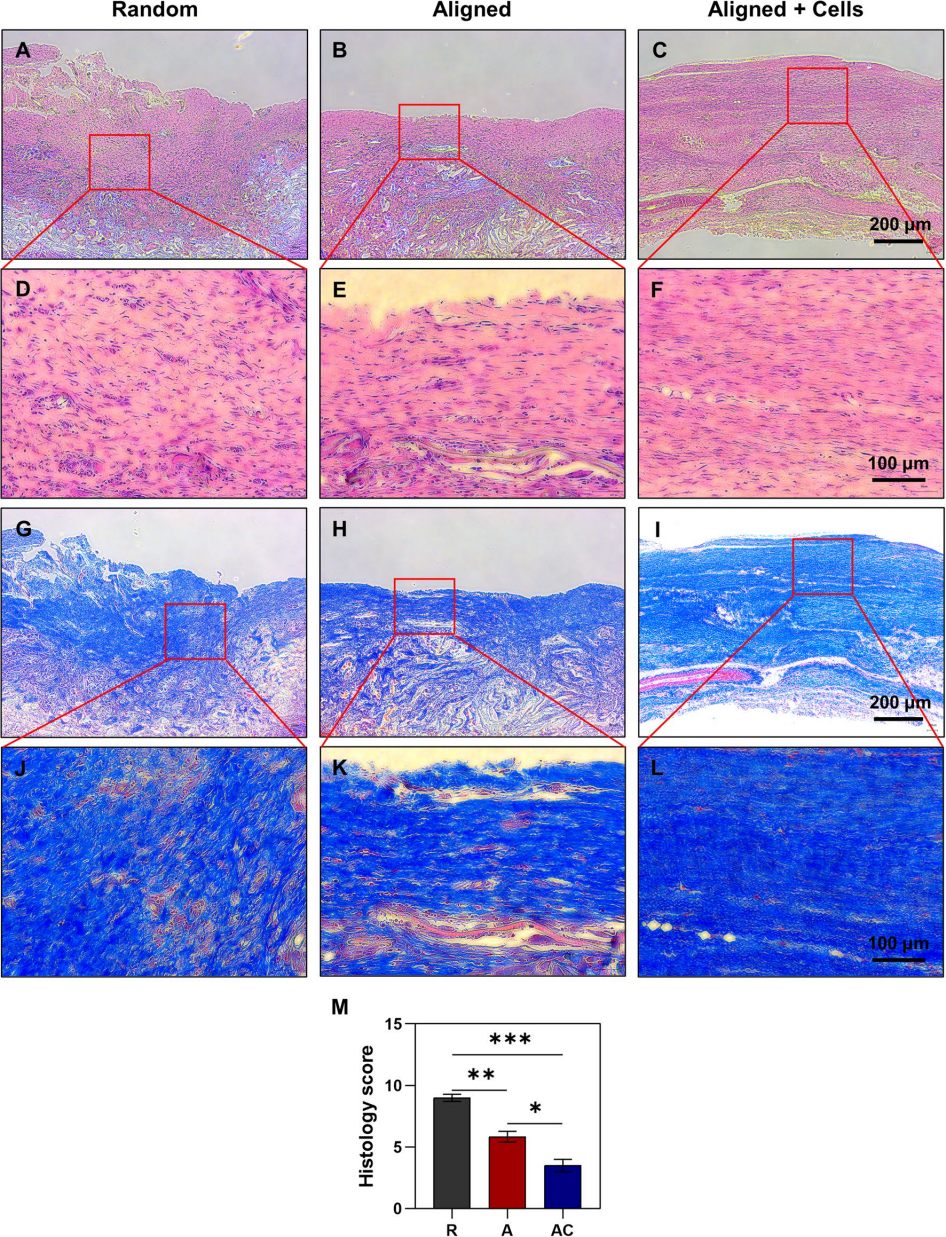
Histological examination of repaired tendons. H&E staining of repaired tissues with different magnifications in Random group (**A**, **D**), Aligned group (**B**, **E**), and Aligned + Cells group (**C**, **F**). Masson trichrome staining of repaired tissues with different magnifications in Random group (**G**, **J**), Aligned group (**H**, **K**), and Aligned + Cells group (**I**, **L**). **M** Histology scores of three groups. *p < 0.05; **p < 0.01; ***p < 0.001

**Fig. 6:**
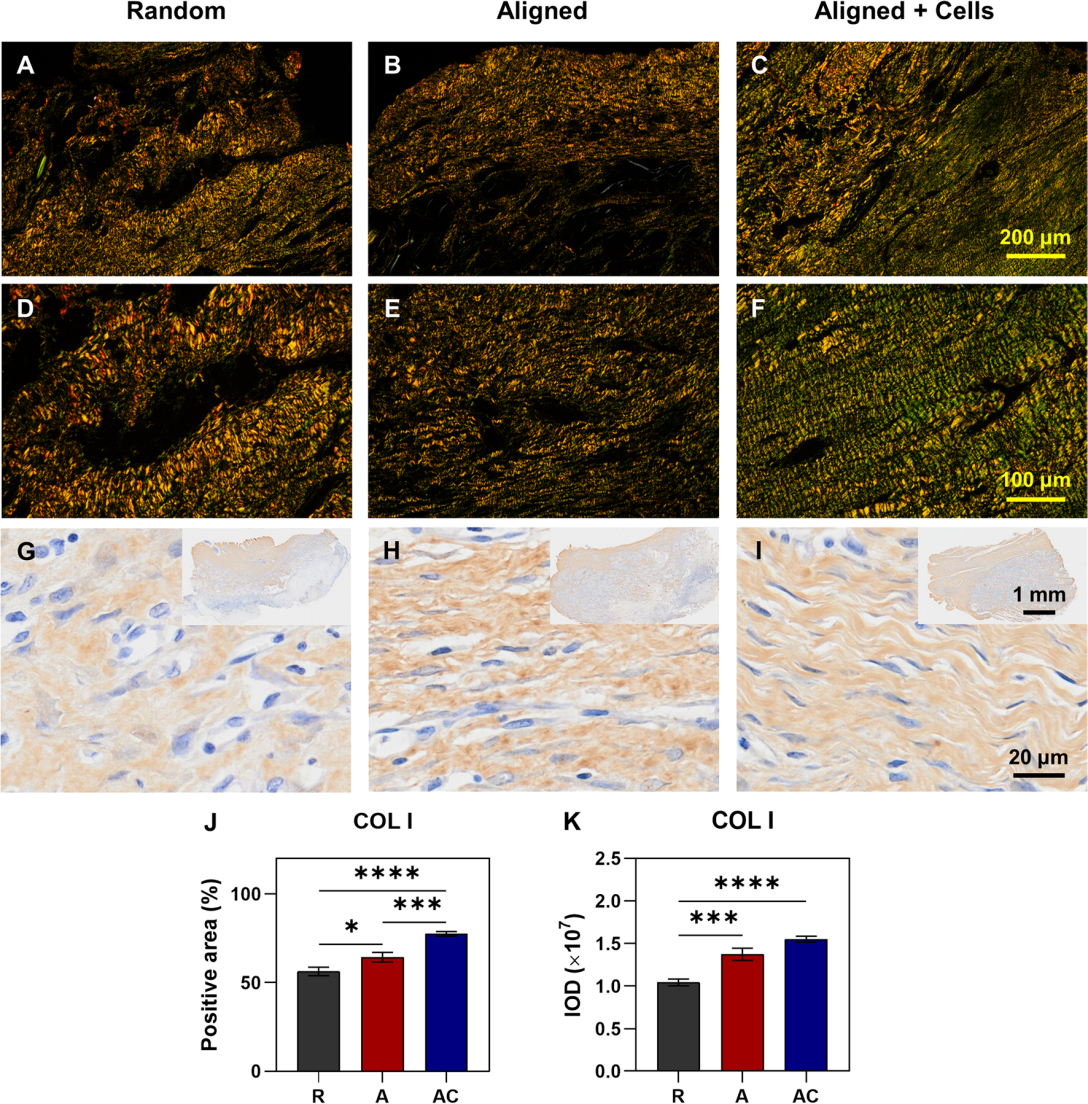
Evaluation of deposited collagen in the repaired tendons. **A**–**F** Polarized light examination of repaired tissues with different magnifications. **G**–**I** Immunohistochemical staining of COL I in the repaired tendons. The upper right panels indicate the images of lower magnifications. **J** The percentage of COL I positive area. **K** Integrated optical density (IOD) values. R group, random silk scaffold alone. A group, aligned silk scaffold alone. AC group, hPDLSCs seeded aligned silk scaffold. *p < 0.05; ***p < 0.001; ****p < 0.0001

To evaluate the roles of hPDLSCs and aligned silk scaffold for tendon tissue engineering in vivo, cells were seeded on aligned silk scaffold and transplanted into the ruptured rat Achilles tendon, with non-cell seeded aligned and random scaffolds as controls (Fig. 4A). After 4 weeks, macroscopical observation found that cell-seeded aligned scaffold (AC group) possessed the best appearance in the defect area, as compared to the aligned scaffold alone (A group, mild scar formation) and random scaffold alone (R group, severe scar formation) (Fig. 4A). The survival of implanted hPDLSCs was confirmed by immunohistochemistry with anti-human nuclei monoclonal antibody (Fig. 4B, red arrows). Further evaluation of repair effect by H&E staining showed denser and more homogenous tendon-like tissue formation in the AC group, with spindle-shaped cells aligned directed, as compared to the scaffold only groups (Fig. 5A–F). Consistently, Masson trichrome staining found denser and more homogenous deposited collagen in the AC group (Fig. 5G–L). The quantified histology scores supported that the AC group possessed a better score (3.50 ± 0.87) than the A group (5.83 ± 0.76) and the R group (9.00 ± 0.50) (Fig. 5M). A significant difference was also found between the group of aligned silk scaffold and random silk scaffold, which indicated the promotion effect of the aligned scaffold itself on tendon repair. The collagen deposition was further evaluated by polarized light examination (Fig. 6A–F). As expected, the collagen fibres in the AC group were more aligned and distributed more evenly than those in the R group (Fig. 6A, C, D, F). Regularly arranged collagens were also found in the A group. However, the deposited collagens were not as homogenous as those found in the AC group (Fig. 6B, C, E, F). Nevertheless, fragmented collagen fibres found in the AC group indicated that the repaired tendons were still not as good as normal ones. COL I is the main type of collagen in normal tendons. The deposition of COL I is therefore detected by immunohistochemistry (Fig. 6G–I). A significant higher positive area ratio was found in the AC group (77.42 ± 5.35%), as compared to the A group (64.29 ± 10.61%) and R group (56.22 ± 9.52%) (Fig. 6J). Similar result was shown in the quantified IOD values (Fig. 6K).

### Human PDLSCs and aligned silk scaffold modulated macrophage polarization during tendon repair

**Fig. 7:**
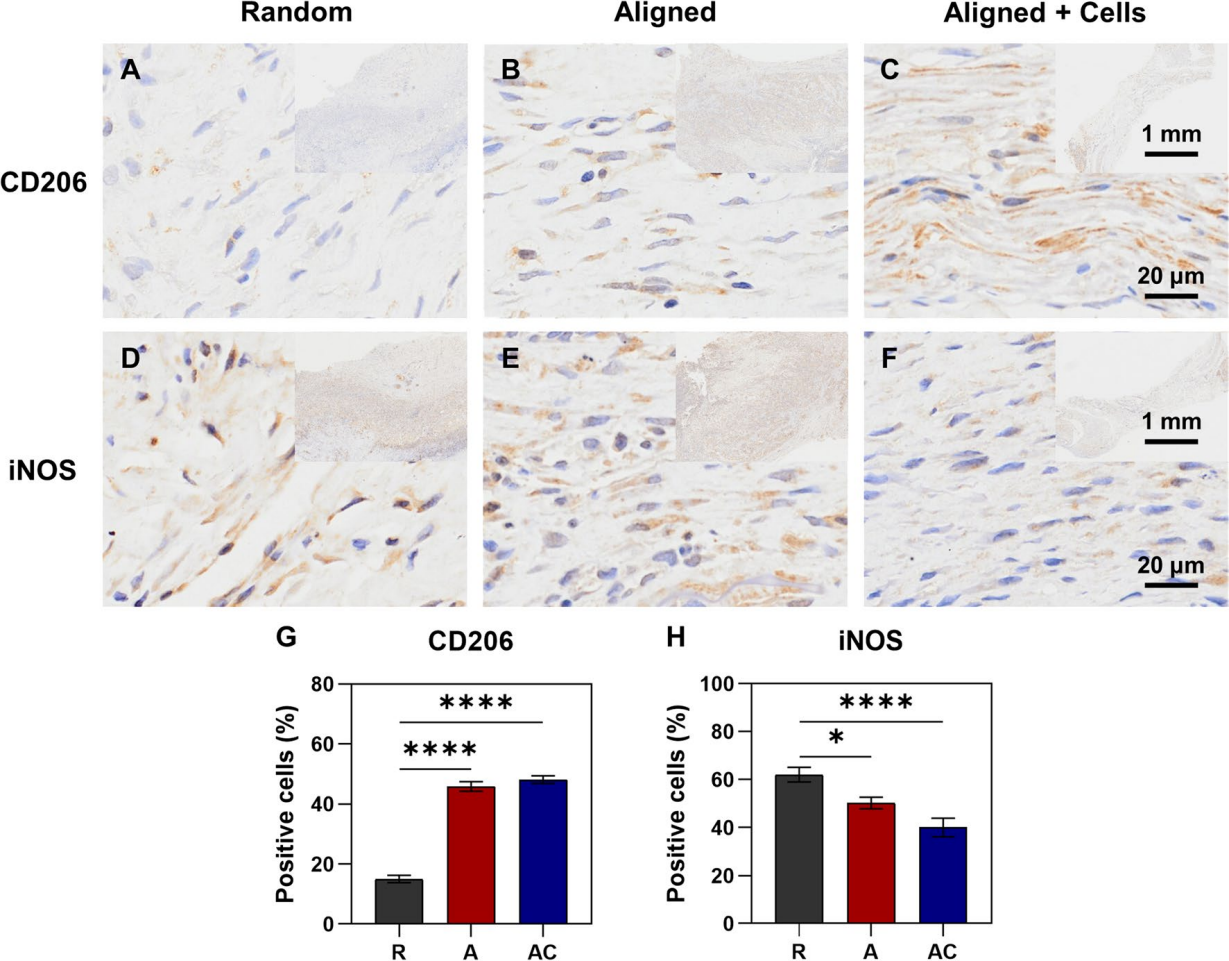
Immunohistochemical staining of macrophage polarization markers CD206 (M2, anti-inflammatory) and iNOS (M1, pro-inflammatory) in the repaired tendons. **A**–**C** Immunohistochemistry with CD206 antibody. **D**–**F** Immunohistochemistry with iNOS antibody. The upper right panels indicate the images of lower magnifications. **G** The percentage of CD206 positive cells. **H** The percentage of iNOS positive cells. R group, random silk scaffold alone. A group, aligned silk scaffold alone. AC group, hPDLSCs seeded aligned silk scaffold. *p < 0.05; ****p < 0.0001

To detect whether hPDLSCs and aligned silk scaffold could influence the phenotype of macrophages during tendon repair, immunohistochemical staining of two typical macrophage polarization markers CD206 (M2, alternatively activated macrophage, anti-inflammatory) and iNOS (M1, classic-activated macrophage, pro-inflammatory) were performed (Fig. 7A–F). Both the hPDLSCs seeded aligned silk scaffold group (48.14 ± 5.04%) and the aligned silk scaffold group (45.87 ± 6.30%) showed significantly higher expression of CD206, as compared to the random silk scaffold group (15.05 ± 4.91%) (Fig. 7G). In contrast, the levels of M1 macrophage phenotype marker iNOS showed opposite results among groups (Fig. 7H).

**Fig. 8:**
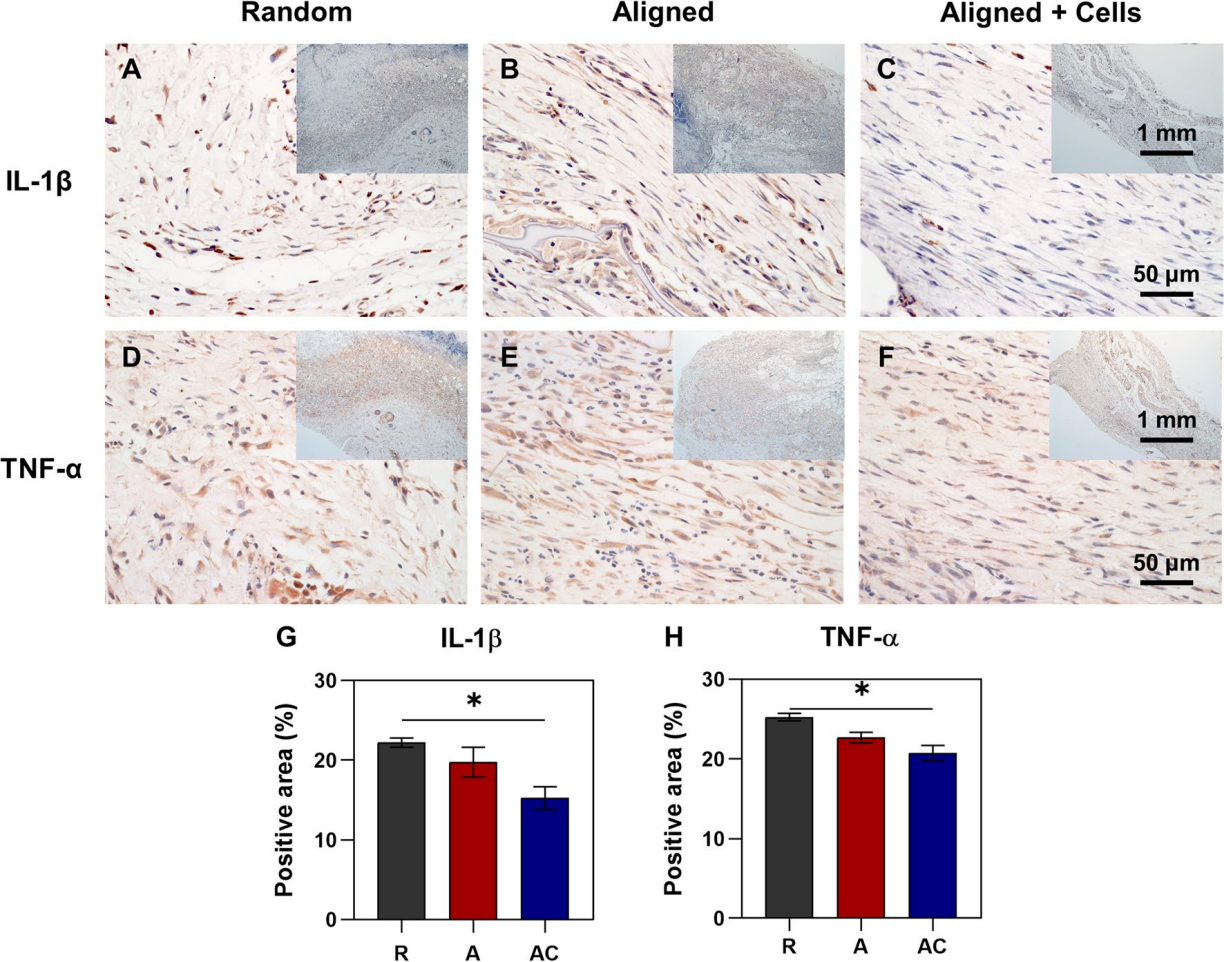
Immunohistochemical staining of inflammatory factors IL-1β and TNF-α in the repaired tendons. **A**–**C** Immunohistochemistry with IL-1β antibody. **D**–**F** Immunohistochemistry with TNF-α antibody. The upper right panels indicate the images of lower magnifications. **G** The percentage of IL-1β positive area. **H** The percentage of TNF-α positive area. R group, random silk scaffold alone. A group, aligned silk scaffold alone. AC group, hPDLSCs seeded aligned silk scaffold. *p < 0.05

Consistent with the changes in macrophage polarization, the hPDLSCs seeded aligned silk scaffold group showed significantly lower expression of pro-inflammatory factor IL-1β (15.25 ± 2.87%) and TNF-α (20.75 ± 2.66%) as compared to the random silk scaffold group (22.20 ± 1.30% and 25.25 ± 0.96%, respectively) (Fig. 8). Though no significant difference was found between any other two groups, the expression levels of pro-inflammatory factors in the aligned silk scaffold were between the other two groups.

## Discussion

This study constructed a tissue-engineered tendon by using human PDLSCs as seeded cells and aligned silk sheet as the tendon-mimetic scaffold. Human PDLSCs were successfully isolated from medical waste (i.e., extracted wisdom teeth), and their multi-differentiation potential was characterized. Aligned silk scaffold was fabricated, and its topography was assessed, as well as its hydrophilicity, degradation and biocompatibility. After being transplanted into the ruptured rat Achilles tendon, hPDLSCs seeded aligned silk scaffold promoted tendon repair with more tendon-like tissue formation, as compared to the scaffold-only groups (aligned or random silk scaffold only). The shift of macrophage polarization status from M1 to M2 phenotype was found in the hPDLSCs seeded aligned silk scaffold group, which contributed to the promotion effect of this combination on tendon repair. Therefore, in this study, the efficacy of hPDLSCs as seed cells, and aligned silk sheet as a scaffold for tendon tissue engineering was evaluated in an in vivo animal tendon rupture model. The results confirmed the application potential of PDLSCs and aligned silk scaffold for tendon repair and regeneration.

Although stem cells derived from bone marrow (BMSCs), adipose tissue (ASCs) and tendon/ligament tissues (TDSCs) have shown their efficacy as seed cells in tendon tissue engineering [27, 28], PDLSCs could be another good choice in spite that their potential in promoting tendon repair and regeneration should be further explored. To be a seed cell for tissue engineering application, the first important concern is accessibility. Some issues should be considered here such as (1) whether the source tissues are abundant to obtain; (2) are there possible ethical problems in collecting the source tissues; (3) will the harvest of source tissues brings additional pain or possible adverse events. With regards to hPDLSCs, it can answer these questions perfectly. Human PDLSCs could be isolated from periodontal ligaments of surgically extracted teeth. As we know, wisdom teeth extraction surgeries are performed worldwide. According to the data from American Dental Association in 1999, there were 10 million wisdom teeth extracted from 5 million people each year in the USA[29]. With the advancement of people’s minds and the increase in medical level, more and more wisdom teeth extraction surgeries are performed annually. Importantly, similar to BMSCs, PDLSCs have an immunomodulatory function and low immunogenicity [7, 8], which make the allogeneic transplantation of PDLSCs practicable. Therefore, by the construction of a “teeth bank”, it is possible to fulfil the application of these cells for future regenerative medicine. What’s more, the extracted teeth are trashed as medical waste traditionally, which therefore decreases the ethical issues to the minimum. Besides, since PDLSCs are isolated from extracted teeth, it is therefore noninvasive and will not bring additional pain or possible adverse events. In comparison, BMSCs are not as accessible as PDLSCs due to the relative difficulty in the collection of bone marrow, which is also invasive and will bring additional pain. TDSCs (besides of PDLSCs) have the same circumstance in this context when the practical application in clinics is taken into consideration.

Robust cell amplification in vitro and tendon differentiation potential are other two characteristics that need to be possessed by ideal seed cells for tendon tissue engineering. Eleuterio et al. noticed that human PDLSCs had a higher proliferation rate than that of BMSCs and dental pulp stem cells (DPSCs) [30], which is important for future clinical application in the regards of time consumption to obtain enough number of cells in vitro. Similar to BMSCs, PDLSCs were found to have the capacity of differentiation towards osteo-, chondro- and adipo-lineage. Our previous study demonstrated the tendon differentiation potential of hPDLSCs in a cell-sheet model. Significantly increased expression of tendon-related genes was found, including *MKX*, *COL I*, collagen type XIV (*COL XIV*), *EPHA4* and *NFATC4* [11]. Moshaverinia et al. compared the capacity for tendon regeneration among human PDLSCs, BMSCs, and gingival mesenchymal stem cells (GMSCs) [13]. Their results revealed that hPDLSCs expressed the highest tendon-related markers including *SCX*, Tenomodulin (*TNMD*), Decorin (*DCN*), and *BGN*. What’s more, in an ectopic animal model of transplanting MSCs subcutaneously, histological and immunohistochemical staining confirmed the superior potential of PDLSCs for tendon regeneration as compared to BMSCs.

Immunoreaction is always one important concern when cells and/or scaffolds need to be transplanted into the defect tissues. Similar to BMSCs, hPDLSCs have low immunogenicity. What’s more, the immunomodulatory function of hPDLSCs has been found a long time ago [8]. Wada et al. found that hPDLSCs inhibited the proliferation of peripheral blood mononuclear cell (PBMC), which was either pre-stimulated with mitogen or in an allogeneic mixed lymphocyte reaction (MLR) [8]. Recently, macrophage polarization during the process of tissue repair is increasingly investigated. Studies show that the shift of macrophage polarization status from M1 to M2 phenotype is quite important for enhanced tissue regeneration [31]. In a periodontal regeneration study, Liu et al. found PDLSCs induced macrophage polarization towards the M2 phenotype by increasing CD206 level, decreasing iNOS and TNF-α level, which contributed to the enhanced periodontal repair after cell transplantation [32]. The immunomodulatory properties of PDLSCs were further confirmed in our current repair of rat Achilles tendons. The transplantation of hPDLSCs decreased pro-inflammatory M1-like phenotype (downregulated iNOS, IL-1β and TNF-α) and increased anti-inflammatory M2-like phenotype (upregulated CD206) in the repaired tendons (Figs. 7 and 8). The experimental group with hPDLSCs transplanted also exhibited the best macroscopical appearance in the defect area after healing (Fig. 4A). Therefore, these promising results further light the future clinical application of hPDLSCs for tendon tissue engineering and tendon regeneration.

The ability of biomaterials on the regulation of inflammatory response was increasingly studied in recent years, which could be determined by various characteristics of scaffolds such as structure, chemical composition, surface roughness and mechanical properties [33]. A study from Schoenenberger et al. revealed the regulation effect of substrate topography on macrophage polarization and inflammatory. Decreased expression of IL-1β was found when cells were cultured on aligned PCL scaffold, as compared to those cultured on random PCL scaffold [34]. Wu et al. compared the immune responses of two biomaterials (silk and polypropylene) after implantation and found that silk induced less recruitment of neutrophils and macrophages, less fibrosis formation, and better tissue healing [35]. In our current study, the biomimetic silk scaffold with aligned structure significantly increased the expression of anti-inflammatory phenotype marker CD206, as compared to the random silk scaffold (Fig. 7G). Moderate decreased expression of IL-1β, TNF-α and iNOS in the repair of ruptured tendon was also found in the biomimetic silk scaffold (Figs. 7 and 8). Better macroscopical appearance in the defect area after healing was also found in the group of aligned silk scaffold (Fig. 4A). These findings are consistent with previous studies [34, 35] and indicate the potential of aligned silk scaffolds in tendon tissue engineering.

Although the results of the current study confirmed the application potential of PDLSCs and aligned silk scaffold for tendon repair and regeneration, there are some limitations. The lack of mechanical evaluation of repaired tendons and the relatively small number of samples weaken the conclusion to some extent. Moreover, the molecular mechanism triggered by PDLSCs and/or aligned silk scaffold has not yet been elucidated. Direct comparisons between PDLSCs and other cell types such as BMSCs, ASCs and TDSCs are also necessary to find the ideal seed cells for tendon tissue engineering. These need to be addressed in the following work.

## Conclusions

Collectively, this study evaluated the efficacy of hPDLSCs as seed cells and aligned silk sheet as the tendon-mimetic scaffold for tendon tissue engineering in an in vivo animal tendon rupture model. The combination of hPDLSCs and aligned silk scaffold promoted tendon repair with more tendon-like tissue formation and modulated the macrophage polarization from M1 to M2 phenotype. As seed cells for tendon tissue engineering, hPDLSCs possess the advantages of accessibility, robust cell amplification ability, high tendon differentiation potential, and appropriate immunomodulatory function. The aligned silk scaffold provided a tendon ECM-like structure and also showed its promotion effect on tendon repair in this study. Therefore, hPDLSC could be a candidate for ideal seed cell for tendon tissue engineering and the combination of hPDLSCs and aligned silk scaffold could promote tendon repair and regeneration.

## Supplementary Information

**Additional file 1: Table S1.** Primers used for qPCR. **Figure S1.** Tenogenic differentiation of hPDLSCs cultured on silk scaffolds. Human PDLSCs were cultured on random and aligned silk scaffolds for 7 days. Gene expression of tendon-related genes was evaluated by qPCR. Expression levels of the random group were set as 1 in the quantified data. No significant difference was found (p ≥ 0.05).

## Data Availability

The datasets used and/or analysed during the current study are available from the corresponding author on reasonable request.

## Abbreviations

hPDLSCs: Human periodontal ligament stem cells
ECM: Extracellular matrix
BMSCs: Bone marrow MSCs
GMSCs: Gingival mesenchymal stem cells
PLA: Polylactic acid
PCL: Poly(ε-caprolactone)
ALP: Alkaline phosphatase
ARS: Alizarin red S
SEM: Scanning electron microscopy
CCK-8: Cell Counting KIT-8
A2-P: L-ascorbic acid 2-phosphate
COL I: Collagen type I
DAPI: 4',6-Diamidino-2-phenylindole
H&E: Haematoxylin and eosin
iNOS: Inducible nitric oxide synthase
IOD: Integrated optical density
SCX: Scleraxis
MKX: Mohawk
EPHA4: EPH receptor A4
NFATC4: Nuclear factor of activated T-cells 4
BGN: Biglycan
M2: Alternatively activated macrophage
M1: Classic-activated macrophage
ASCs: Stem cells derived from adipose tissue
TDSCs: Stem cells derived from tendon/ligament tissues
DPSCs: Dental pulp stem cells
COL XIV: Collagen type XIV
TNMD: Tenomodulin
DCN: Decorin
PBMC: Peripheral blood mononuclear cell
MLR: Mixed lymphocyte reaction
