# Supplementary material for: The application of human periodontal ligament stem cells and biomimetic silk scaffold for in situ tendon regeneration
Source: Stem Cell Res Ther. 2021 Dec 4;12:596. doi: 10.1186/s13287-021-02661-7 (PMC8642874; doi:10.1186/s13287-021-02661-7)
Supplement: Supplementary file 1 — Additional file 1: Table S1. Primers used for qPCR. Figure S1. Tenogenic differentiation of hPDLSCs cultured on silk scaffolds. Human PDLSCs were cultured on random and aligned silk scaffolds for 7 days. Gene expression of tendon-related genes was evaluated by qPCR. Expression levels of the random group were set as 1 in the quantified data. No significant difference was found (p ≥ 0.05). [file 13287_2021_2661_MOESM1_ESM.pdf]

The application of human periodontal ligament stem cells and biomimetic silk scaffold for in situ tendon regeneration

Jialin Chen[a,b,c,*]; Qingyun Mo[a]; Renwang Sheng[a]; Aijing Zhu[a]; Chen Ling[d]; Yifan Luo[a]; Aini Zhang[a]; Zhixuan Chen[a]; Qingqiang Yao[c,d]; Zhuoying Cai[e,*]; Wei Zhang[a,b,c,*]

[a] School of Medicine, Southeast University, Nanjing, 210009, China

[b] Jiangsu Key Laboratory for Biomaterials and Devices, Southeast University, 210096, Nanjing, China

[c] China Orthopedic Regenerative Medicine Group (CORMed), China

[d] Department of Orthopaedic Surgery, Institute of Digital Medicine, Nanjing First Hospital, Nanjing Medical University, 210006, Nanjing, China

[e] Oral and Maxillofacial Surgery, the Second Affiliated Hospital of Zhejiang University, 310009, Hangzhou, China

Supplementary methods

Tenogenic differentiation of hPDLSCs cultured on silk scaffolds

Human PDLSCs were seeded on random and aligned silk scaffolds, and cultured in high glucose DMEM (Gibco, C11995500) supplemented with 10% fetal bovine serum (Wisent, 086-550), 1% penicillin-streptomycin (Gibco, 15140122) and 50 μg/ml L-ascorbic acid 2-phosphate (A2-P; Sigma-Aldrich, A8960). After 7 days of culture, RNA extraction (Tiangen, DP430), cDNA reverse transcription (Toyobo, FSQ-201) and quantitative polymerase chain reaction (qPCR; Agbio, AG11718) were performed according to the manufacturers' protocols. All primers used in this study were summarized in Table S1. Representative results are displayed as target gene expression normalized to housekeeping gene GAPDH.

Table S1. Primers used for qPCR.

Genes	5'-3'	Primers
Glyceraldehyde 3-phosphate	Forward	TGACGCTGGGGCTGGCATTG
dehydrogenase (*GAPDH*)	Reverse	GGCTGGTGGTCCAGGGGTCT
Scleraxis (*SCX*)	Forward	CGAGAACACCCAGCCCAAAC
	Reverse	CTCCGAATCGCAGTCTTTCTGTC
Mohawk (*MKX*)	Forward	GAAGGCAACTTTGTCTATCGCA
	Reverse	TGATCTCCTTCCAATACGTGTC
Collagen type I (*COL I*)	Forward	CGATGGATTCCAGTTCGAGTAT
	Reverse	CATCGACAGTGACGCTGTAGG
EPH receptor A4 (*EPHA4*)	Forward	AGTGGGCTGTGACAATCTGGAATA
	Reverse	CATTTAGACGGAACTGAGGAGGGT
Nuclear factor of activated T-cells 4	Forward	AAGGGTGAGACGGACATCG
(*NFATC4*)	Reverse	CCGCCCATTGGAGACATAA
Biglycan (*BGN*)	Forward	GATGGCCTGAAGCTCAA
	Reverse	GGTTTGTTGAAGAGGCTG

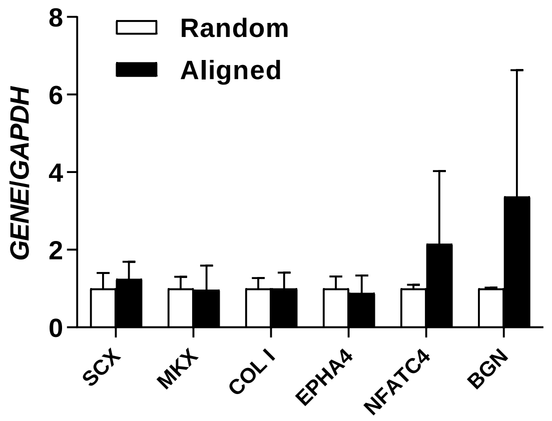

Figure S1. Tenogenic differentiation of hPDLSCs cultured on silk scaffolds. Human PDLSCs were cultured on random and aligned silk scaffolds for 7 days. Gene expression of tendon-related genes was evaluated by qPCR. Expression levels of the random group were set as 1 in the quantified data. No significant difference was found ($p \geq 0.05$).
